# Genomic Tools for the Conservation and Genetic Improvement of a Highly Fragmented Breed—The Ramo Grande Cattle from the Azores

**DOI:** 10.3390/ani10061089

**Published:** 2020-06-24

**Authors:** Andreia J. Amaral, Ana L. Pavão, Luis T. Gama

**Affiliations:** 1 CIISA—Centro de Investigação Interdisciplinar em Sanidade Animal, Faculdade de Medicina Veterinária, Universidade Lisboa, Avenida da Universidade Técnica, 1300-477 Lisboa, Portugal; ltgama@fmv.ulisboa.pt; 2 Direção Regional da Agricultura, Secretaria Regional da Agricultura e Florestas da Região Autónoma dos Açores, Vinha Brava, 9701-861 Angra do Heroísmo, Portugal; Ana.LM.Pavao@azores.gov.pt

**Keywords:** functional genomics, GWAS, inbreeding, local breeds, ROH, single nucleotide polymorphisms (SNPs)

## Abstract

**Simple Summary:**

Inbreeding control is a key concern in managing local endangered breeds, which often have developed unique adaptation features. Ramo Grande is a local cattle breed raised in the Azores archipelago under very harsh conditions, with a census of about 1300 cows dispersed by various islands. This fragmentation is a challenge when the goal is to keep inbreeding under control. Currently, panels of genetic markers are available which enable the assessment of inbreeding and the occurrence of previous bottlenecks in a population. These panels also allow the identification of genes associated with specific production traits, if reliable phenotypic information is available. We used a panel of genetic markers and estimated that the degree of inbreeding was approaching a level of concern, while some exotic gene inflow may have occurred in the past. We were able to identify genetic markers significantly associated with longevity, which reflects the ability of these cattle to remain productive under severe environmental conditions. Genetic markers were also identified as significantly associated with age at first calving and calf growth rate. The results indicate that genomic information can be used to control inbreeding and to implement genomic selection in Ramo Grande cattle to enhance adaptation and production traits.

**Abstract:**

Ramo Grande is a local cattle breed raised in the archipelago of Azores, with a small and dispersed census, where inbreeding control is of utmost importance. A single nucleotide polymorphism (SNP) Beadchip array was used to assess inbreeding, by analysis of genomic regions harboring contiguous homozygous genotypes named runs of homozygosity (ROH), and to estimate past effective population size by analysis of linkage disequilibrium (LD). Genetic markers associated with production traits were also investigated, exploiting the unique genetic and adaptation features of this breed. A total of 639 ROH with length >4 Mb were identified, with mean length of 14.96 Mb. The mean genomic inbreeding was 0.09, and long segments of ROH were common, indicating recent inbred matings. The LD pattern indicates a large effective population size, suggesting the inflow of exotic germplasm in the past. The genome-wide association study identified novel markers significantly affecting longevity, age at first calving and direct genetic effects on calf weight. These results provide the first evidence of the association of longevity with genes related with DNA recognition and repair, and the association of age at first calving with aquaporin proteins, which are known to have a crucial role in reproduction.

## 1. Introduction

The diversity of animal genetic resources plays a crucial role in meeting human needs and is essential for maintaining the efficiency of production systems, particularly as they are threatened by climate change [1]. Maintaining local breeds is thus vital for facing an uncertain future, but many of these breeds have been abandoned or are currently threatened by more competitive cosmopolitan breeds [2].

**Figure 1:**
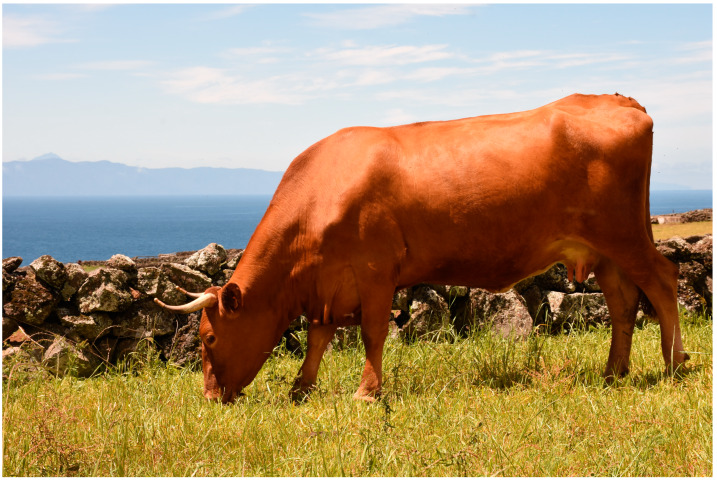
Representative cow of the Ramo Grande breed in the Azores archipelago.

In local breeds, often with small population size, the increase of inbreeding is one of the major concerns, because of the associated loss of genetic diversity and the deleterious impact of inbreeding depression [3]. The Ramo Grande cattle breed (Figure 1) was developed in the Azores islands starting in the 15th century, resulting from a founder population brought by the Portuguese, which may have received additional genetic influences over the centuries. The Azores are a group of volcanic islands located in the middle of the Atlantic, and Ramo Grande cattle currently have a registered census of about 1300 cows and 80 bulls, dispersed by seven of the nine islands that make-up the archipelago. There is reduced gene-flow between islands, except for the use of artificial insemination with semen from a common group of bulls, but some inflow of genes from mainstream breeds may have occurred in recent past [4]. Most likely, this breed was selected over the centuries for adaptation to the unique harsh environment of the Azores, potentially harboring specific genetic variants that are worth conserving and possibly of high value for the future. Currently, the breed is used for meat production and for draught in agricultural activities, but it also plays a significant role in cultural traditions, where Ramo Grande ox pairs often participate in local corteges.

A program aimed at the genetic management of Ramo Grande cattle has been in place for nearly 20 years, essentially based on the collection of phenotypic and pedigree data, with the goal of selecting for some production traits while maintaining inbreeding under control [5]. Still, the estimated effective population size (*N*e) based on pedigree information was 28 [5] which is nearly one-half of the recommendation of the FAO for the maintenance of genetic diversity [6].

The use of medium-density single nucleotide polymorphism (SNP) arrays allows an alternative but accurate estimation of inbreeding (*F*), even in the absence of pedigree information, and provides insight into the levels of linkage disequilibrium (LD) and the corresponding Ne [7]. Currently, the estimation of F is often based on the analysis of runs of homozygosity (*F*_ROH_) [8], which are contiguous lengths of homozygous genotypes that are present in an individual due to the transmission of identical haplotypes from parents to offspring [9]. *F*_ROH_ provides a good indication of individual autozygosity at the genome-wide level and allows the distinction between ancient and recent inbreeding, as recombination tends to disrupt long chromosome segments [10] and long ROH (~10 Mb) thus arise from recent inbreeding. On the other hand, the analysis of LD allows the estimation of *N*e, which is a useful way to evaluate the loss of genetic diversity over time [11].

In addition to conserving genetic diversity of local breeds, the improvement of their productive performance while maintaining adaptability to local environmental conditions is desired, with the goal of making them more competitive and thus self-sustaining. Therefore, identifying polymorphisms in genes that may influence important traits is also crucial for the current management/improvement program of this breed.

Genomic tools developed in recent years may provide insight and a thorough understanding of the threats to genetic diversity of local breeds [12], and reveal genetic variability for traits associated with production ability, including adaptation traits that may be of importance for sustainable agriculture in a changing climate [13]. In this study, we used the 50K v3 Illumina Beadchip to assess the threats to genetic diversity in Ramo Grande, by estimating the occurrence of ROH, as well as the levels of LD and the corresponding Ne in past generations. Moreover, in order to identify polymorphisms significantly associated with relevant traits, we used a genome-wide association analysis, and investigated the functional impact of the identified associations by examining gene-interaction networks, allowing a better understanding of the genetic influences and candidate genes affecting the traits of interest [14].

## 2. Materials and Methods

### 2.1. Samples, Genotypes and Quality Control

Pedigree data has been collected for Ramo Grande cattle since 1982, and currently there are 8931 accumulated records in the Herdbook. DNA samples were obtained from 44 Ramo Grande bulls with semen available for commercial use. These bulls were sampled from several farms located in different islands across the archipelago of Azores (São Jorge, Faial, São Miguel, Graciosa, Pico and Terceira islands). Given the small number of sires existing in the breed, the sampled bulls were inevitably related, with an average relationship among them of 4.12 ± 1.81%.

DNA was extracted from either blood (*n* = 11) or semen (*n* = 33) using QIAsymphony robot (QIAGEN) according to manufacturer instructions. All animals were genotyped with the Bovine SNP50v3 Analysis Beadchip (Illumina Inc. San Diego, CA, USA), obtaining genotypes for about 54K SNPs. Data quality control was performed using PLINK v1.90 [15]. All SNPs not assigned to the *Bos taurus* genome (assembly UMD 3_2), or assigned to chromosomes X and Y were excluded. Markers were then selected according to the following quality criteria: (i) call frequency (≥0.95), minor allele frequency (MAF ≥ 0.01) and Hardy-Weinberg equilibrium (*p*-value > 0.001). SNPs that did not satisfy these criteria were excluded from further analyses. Moreover, considering that LD can lead to the detection of ROH that are not truly identical by descent (IBD), LD pruning was performed before ROH call in order to increase power, as advised by Purcell et al. (2007) [15] and applied by several authors [16,17,18]. Unlinked SNPs were selected using the indep option of PLINK with the following parameters: 50 SNPs/window, a shift of five SNPs between windows and *r*^2^ threshold of 0.5.

### 2.2. Analyses of Runs of Homozygosity and Estimates of Inbreeding

The following criteria were used to define the ROH: (i) sliding window of 15 SNP loci; (ii) minimum number of 40 SNPs for ROH identification; (iii) minimum SNP density of 1/100 kb (1000 bp); (iv) maximum gap between consecutive SNPs of 1 Mb; (v) Minimum thresholds of ROH length of 4 Mb. These were investigated as advised by Ferenčaković et al. (2013) [19] and applied by another study in minor cattle breeds [17]. The estimates of ROH were calculated using the detectRUNS package [20] in R environment [21]. The proportion of ROH segments >4 Mb relative to the full chromosome length was considered as the genomic inbreeding (*F*_ROH_). In addition, to investigate the effect of other criteria in the definition of *F*_ROH_, we also considered *F*_ROH_ as the proportion of ROH segments >8 Mb relative to the full chromosome length [22].

### 2.3. Linkage Disequilibrium and Effective Population Size

The estimate of linkage disequilibrium (*r*^2^) between SNP pairs within the same chromosome was obtained using PLINK [15], by considering *r*^2^ ≥ 0. For assessment of the relationship of LD with genomic distance, the mean LD was computed in bins of 10K. The effective population size was then estimated using LD measurements according to Corbin et al. (2012) [23] using SNeP [24].

### 2.4. Deregression of Estimated Breeding Values

A Herdbook has been in place for the Ramo Grande breed since 1995, where animals are registered after being classified according to morphology and type at around 2 years of age. Animals are scored for nine body conformation traits, and to enter the Herdbook they must receive a total type score of at least 75 in a maximum of 100 possible points. As part of the on-going management program of this breed, phenotypic data has been collected since 1996, including measurement of the following traits:

(i) Type score—total sum of points attributed to various body conformation traits assessed at the time of registration;
(ii) Temperament—also assessed at the time of registration in the Herdbook, where animals are subjectively scored from very docile (score = 1) to unyielding (score = 5);
(iii) Age at first calving—age in days when a cow had its first calf;
(iv) Calving interval—interval in days between two consecutive calvings of a cow;
(v) Number of calvings through life—total number of calvings in the lifetime of a cow;
(vi) Longevity—computed as the age in months at the last registered calving plus 7 months, assuming that the cow lasted at least up to calf weaning;
(vii) Calf weight adjusted to 4 months of age—this weight was assumed to receive both direct and maternal genetic influences;
(viii) Carcass weight per day of age, computed as carcass weight divided by age at slaughter—this growth rate was assumed to receive both direct and maternal genetic influences.

Estimated breeding values (EBV) are routinely predicted for these traits using univariate Animal Models with fixed and random effects specific for each trait. The linear mixed models used for analyzing the various traits were as follows.

(1) Analysis of type score, temperament, age at first calving, longevity and number of calvings:

y = Xb + Z_a_a + e (1)

(2) Analysis of calving interval:

y = Xb + Z_a_a + Z_p_p + e (2)

(3) Analysis of live weight at 4 months and carcass weight/day of age:

y = Xb + Z_a_a + Z_m_m + Z_p_p + e (3)

where:

‘y’ is the vector of observations;

‘b’ is the vector o fixed effects;

‘a’ is the vector of additive direct genetic effects;

‘m’ is the vector of additive maternal genetic effects;

‘p’ is the vector of permanent maternal environmental effects;

‘e’ is the vector of residual effects;

‘X, Z_a_, Z_m_, e, Z_p_’ are known incidence matrices relating the fixed effects (X) and each of the random effects (Z_a_, Z_m_, e, Z_p_) with the vector of observations ‘y’.

Variance components were estimated and breeding values predicted with the BLUPF90 package [25].

For the genome-wide association analyses, EBVs of the genotyped bulls were obtained from the published annual evaluations, which are based on phenotypic records collected on an average of 55.5 ± 91.3 offspring per bull. These EBVs were then used to compute deregressed EBV (DEBV), calculated by applying the procedure described by Garrick et al. (2009) [26], considering that the fraction of genetic variance not explained by markers (c) was 0.10. The deregression procedure of Garrick et al. (2009) [26] takes into consideration the estimated reliability of the EBV, thus accounting for the amount of information available on relatives of the evaluated individuals, which in our case included phenotypic records on a total of 2332 offspring of the genotyped bulls. The deregression step also eliminates shrinkage inherent to the computation of EBV, and therefore deregressed EBV behave as though they were observations with a heritability equal to the reliability of the deregressed EBV [27].

### 2.5. Association Analysis

Using the obtained DEBV as phenotypes, single SNP genome-wide association analyses (GWAS) were performed using the GenABEL toolset, accounting for stratification using the principal component analysis method with the *egscore* function [28]. The reliability of the detected associations was evaluated by investigating the deviation from the null distribution through the generation of quantile-quantile (Q-Q) plots in R environment [21]. The *p*-values were then corrected by multiplying observed *p*-values by the deflation factor λ obtained in GWAS [29]. We used the two higher level genome-wide significance thresholds defined by Teyssedre et al. (2012) [30] and used by [14,29,31]. The used thresholds were 5 × 10^−6^ and 5 × 10^−5^, where the first corresponds to 10,000 independent Bonferroni corrected tests and the second was proposed as the threshold allowing the detection of moderate associations [30]. The proportion of variance explained by each significantly associated SNP was estimated according to the method described by [32]

### 2.6. Candidate Genes and Functional Analysis

Putative candidate genes overlapping significantly associated SNPs for the studied traits were identified based on the ARS-UCD1.2 assembly, using the NCBI Map Viewer (http://www.ncbi.nlm.nih.gov/projects/mapview/). The Genemania package [33] of Cytoscape 3.4.0 was then used to generate gene interaction networks, allowing to identify genes functionally related with the candidate genes. This was carried out based on the information reported for humans and only cattle orthologous genes were considered for the networks. The Reactome database was used (www.reactome.org) to perform analyses of pathway enrichment as described in [14].

## 3. Results

The main objectives of this study were to estimate the level of inbreeding in the Ramo Grande endangered cattle breed using genomic information, and to identify SNPs significantly associated with relevant economic traits currently selected for. This is the first characterization of this breed using medium-density SNP arrays, and should provide useful information and novel guidelines for a better management of genetic diversity and selection for efficiency in this fragmented breed.

### 3.1. Genotype Quality Control

Following the genotype control stage, starting from 54,609 markers, 1568 variants were removed due to missing genotype data and 14,119 variants were removed due to minor allele threshold. With respect to samples, two individuals were removed because of low call rate (<0.95). These criteria resulted in a set for LD and GWAS analyses comprising 37,727 markers in 42 animals. On the other hand, for ROH estimates we further prepared a subset of genotypes that was processed for LD pruning, which included 21,884 SNPs.

### 3.2. ROH Estimates

Using the subset of data that was pruned for SNPs in LD, we have performed the identification of ROH segments in order to estimate the inbreeding level in this breed. Using the criterion of one ROH being at least 4 Mb long, a total of 639 ROH were identified, and all individuals displayed at least one ROH segment. As shown in Table 1, the frequency was similar for segments of small (4–8 Mb), intermediate (>8–16 Mb), and large (>16 Mb) size.

**Figure 2:**
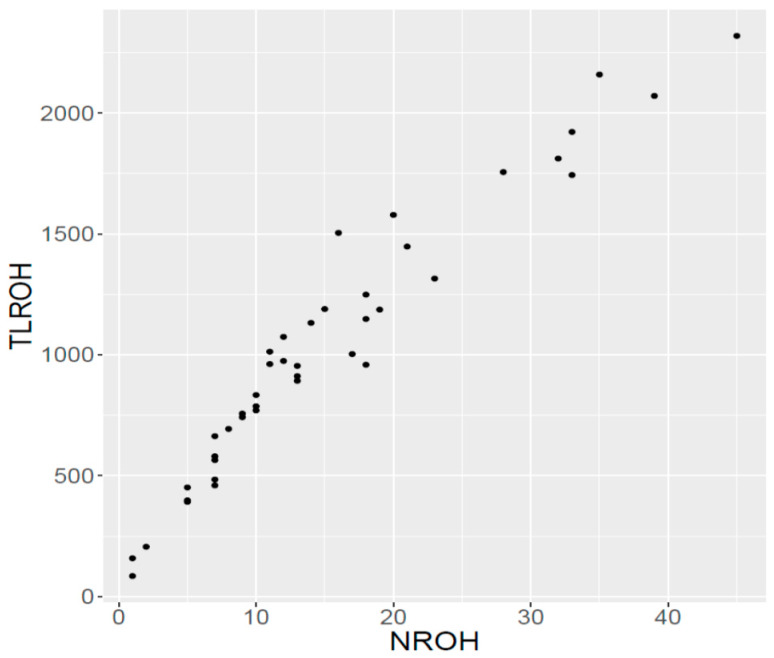
Relationship between total number of ROH (NROH) and total length of the ROH (TLROH, in Mb) per animal, for Ramo Grande bulls. Each dot represents an individual.

The mean number of ROH by chromosome was 15.2 (Table 2) and the chromosome that displayed the higher number of ROH segments was BTA6, followed by BTA4 and BTA5 (Table S1). The chromosome that displayed the largest mean for ROH length was BTA8, where some of the segments reached 50 Mb (Table S2). Overall, the maximum size of ROH (Table 2) was 78.30 Mb and it was observed in BTA3. As shown in Figure 2, the number of ROH and the length of these fragments per individual had a strong positive correlation.

**Figure 3:**
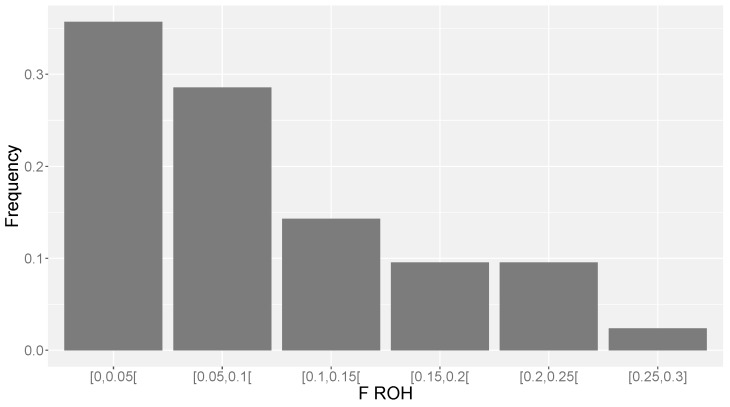
Distribution of *F*_ROH_ estimates across animals.

The mean estimate of *F*_ROH_ per individual was 0.091 ± 0.073 when a minimum threshold of 4 Mb was used (Table 2), whereas if we considered a minimum length of 8 Mb we obtained a mean value of *F*_ROH_ = 0.080 ± 0.068 (results not shown). Overall, the variability in *F*_ROH_ between animals was large (Figure 3), with nearly one-third of the animals with an *F*_ROH_ above 0.15.

### 3.3. Linkage Disequilibrium Decay and Ancestral Effective Population Size

**Figure 4:**
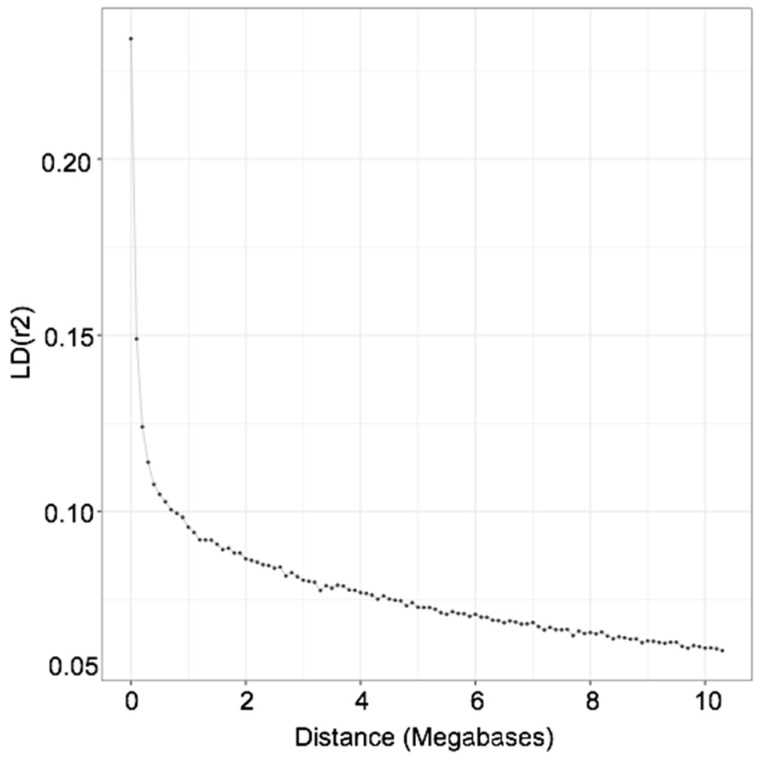
Linkage disequilibrium across the genome as a function of genomic distance (Mb) for Ramo Grande cattle.

**Figure 5:**
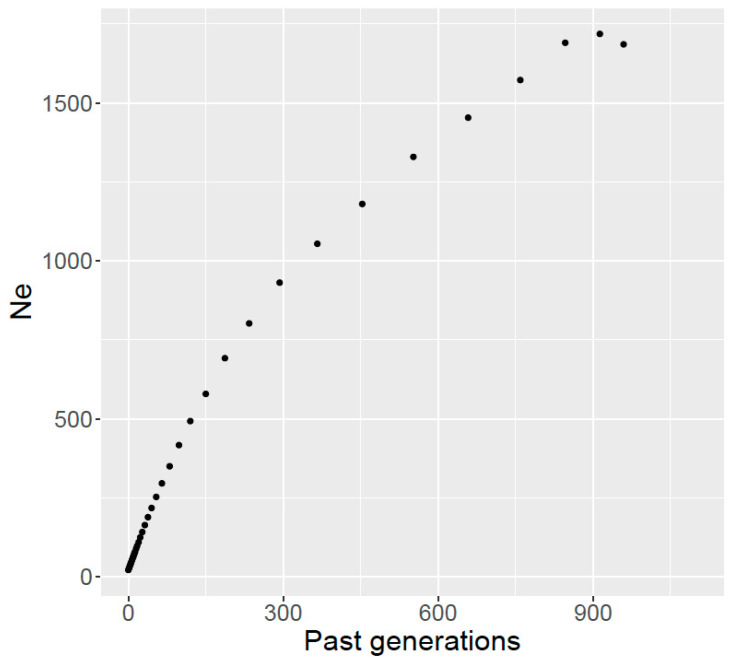
Estimation of ancestral effective population size (Ne) in past generations, estimated from linkage disequilibrium.

The analysis of linkage disequilibrium (LD) decay can contribute to better understand the extent of ROH as well as to enable the estimation of effective population size in breeds for which the pedigree information is not very deep or is unreliable. Figure 4 shows the relationship between levels of LD (average pairwise *r*^2^ values) and distance between markers, for SNPs located on the same chromosome and estimated using the GWAS subset of markers. We observed that for distances of 1 Mb the *r*^2^ was about 0.1 while for a distance of 4 Mb the values of *r*^2^ were about 0.075. The estimation of ancestral effective population size from LD decay indicates that 5 generations ago the effective population size of Ramo Grande was 44 animals (Figure 5). We also observed a nearly linear relationship between number of generations and effective population size, in which effective population size increased with the increase in number of generations, with an effective population size estimated at more than 400 individuals 100 generations ago.

### 3.4. Genome-Wide Association Analyses and Candidate Genes

In the selection program of Ramo Grande, a single-trait genetic evaluation is carried-out yearly for several production traits (see methods). The descriptive statistics and genetic parameters estimated by REML for the various traits are in Table 3. The highest estimated heritabilities were observed for type score and temperament and for direct genetic effects on carcass weight/day of age and on weight at 4 months.

**Figure 6:**
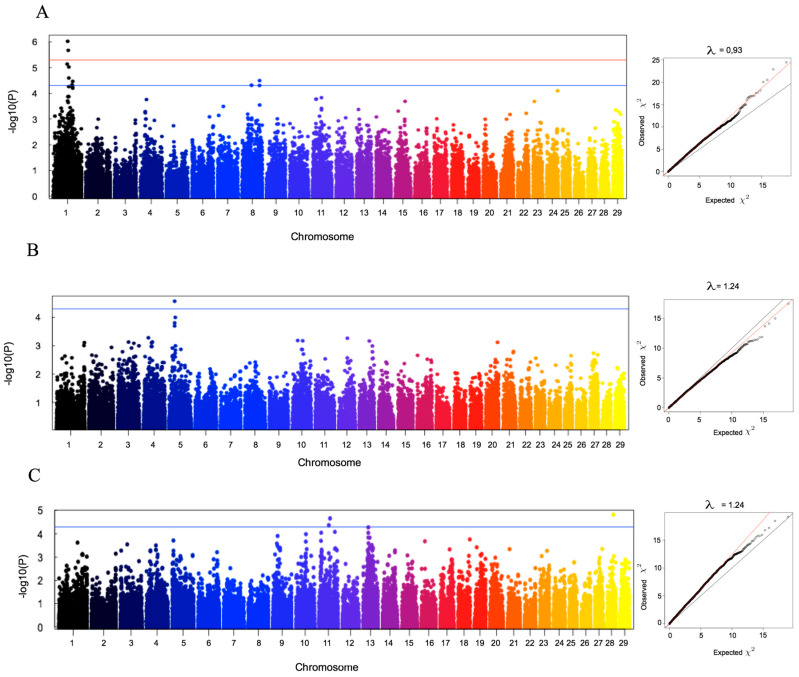
Manhattan plot for single SNP GWAS with correction for population structure. (**A**) Longevity; (**B**) age at first calving; (**C**) direct genetic effect for calf weight at 4 months. The red and blue horizontal lines correspond to genome-wide significant and suggestive thresholds, namely adjusted-*p*-value = 0.000005 and adjusted-*p*-value = 0.00005, respectively. Q-Q plots generated to assess the reliability of the detected associations are shown for each analyzed trait along with the corresponding deflation factor (λ).

We have investigated the presence of significant associations between SNPs and the estimated DEBV for these traits. The association testing was conducted accounting for population stratification using the principal component analysis method, where the egscore function and *p*-values were then corrected by multiplying observed *p*-values by the deflation factor λ obtained in GWAS (see methods). We observed strong significant associations for three of the studied traits, i.e., longevity, age at first calving and direct genetic effect on calf weight at 4 months (Table 4, Figure 6), with the most significant associations observed for longevity. Some of the SNPs significantly associated are located in intronic regions, or in regions involved in the regulation of the expression of nearby genes.

### 3.5. Significant Associations with Longevity

We have identified significant associations of longevity with SNPs located in BTA1 and BTA8 (Figure 5A, Table 4). The BTA1 is where we observed the most significant association with a SNP located in upstream of gene *GMNC* that encodes the Geminin coiled-coil domain containing protein. This signal is supported by the significant association of other SNPs located nearby in intergenic regions, creating a typical pattern of a bona fide genotype-phenotype association with a level of significance that exceeds the highest significant threshold (0.000005). The other observed significant associations for this trait where we could infer functionality are located in BTA8, upstream of *PRUNE2* that encodes for protein homolog2, and in the intron region of *RAD23B* gene, that encodes for the ubiquitin protein receptor *RAD23B*.

**Figure 7:**
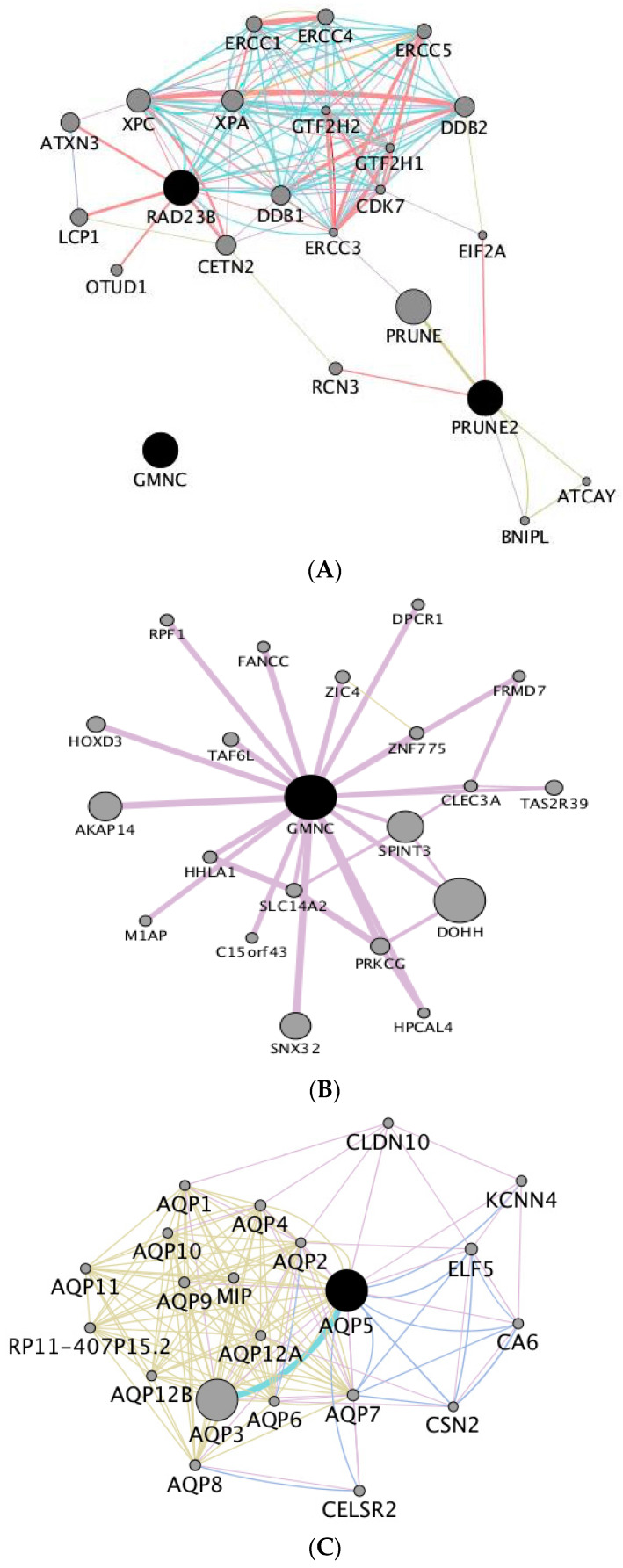

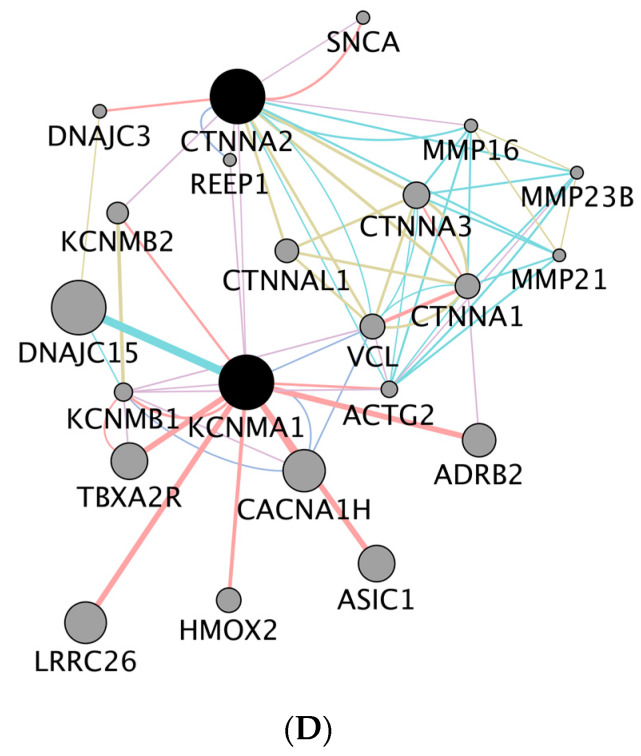
Gene networks constructed based on the candidate genes overlapping significant SNPs for the studied traits. (**A**) Genes associated with longevity; (**B**) *GMNC* interaction network; (**C**) genes associated with age at first calving; (**D**) genes associated with direct genetic effects for calf weight at four months. Circles represent network nodes connected by lines (edges). The size of the nodes depends on their level of connectivity in the network (as more edges result in a larger circle size). On the other hand, edges with thicker line weights correspond to gene-gene interactions with stronger support by the literature.

In a second stage, we investigated the relationships between these genes, by computing the corresponding gene interaction network. We observed that *PRUNE2* and *RAD23B* have strong interactions, and do not display connections with *GMNC* (Figure 7A). Therefore, we have investigated separately the network genes interacting with *GMNC* (Figure 7B). The network-based analysis of pathway enrichment revealed a high representation of genes related with DNA damage recognition, global genome nucleotide excision repair and regulation of gene expression (Table 5).

### 3.6. Significant Associations with Age at First Calving and with Direct Genetic Effects for Calf Weight at Four Months

The association testing between DEBV for age at first calving and SNPs, allowed identifying a significant association with a SNP located in BTA5 (Table 3) which is upstream of gene *AQP5*, that encodes aquaporin 5, which is a water channel protein. This gene interacts with other genes encoding other aquaporin forms with a similar function and several other genes, in a total of 20 genes. The network-based gene set enrichment analysis (Figure 7C) allowed us to observe an enrichment of biological processes related with pathways of transport of small molecules and signal transduction (Table 6).

We have further identified significant associations between the DEBV for direct genetic effects for calf weight at four months and SNPs located in introns of two genes, specifically *KCNMA1* (Potassium Calcium-Activated Channel Subfamily M Alpha 1) and *CTNNA2* (Catenin Alpha 2). These genes are pivots of a gene network composed by two hubs (Figure 7D), and this network is enriched in biological processes related with signal transduction, homeostasis disease metabolism of proteins and neuronal system (Table 7).

## 4. Discussion

In this study we have performed the first genome-wide characterization of the Ramo Grande cattle breed, including assessment of inbreeding and identification of genetic markers associated with production traits. This local breed from the Azores archipelago is characterized by a small population census, spread by various islands, and very well adapted to local conditions unique to the volcanic territory where it has evolved. Also unique to this breed is the cultural role that it plays in local folklore, specifically by the participation of ox pairs in corteges organized according to customary traditions.

The results of genotyping using the Illumina 50K SNP chip showed that only about 68% of the assayed SNPs are polymorphic for this breed, which is in accordance with other studies with local cattle breeds that were not included in the SNP chip discovery panel [14,17], and indicate that ascertainment bias may be a concern when SNP chips are applied to minor breeds.

The level and time of occurrence of inbreeding was assessed by the extent of ROH segments in the sampled group [10], as it has been shown that a strong relationship exists between pedigree and genomic inbreeding, even though pedigree inbreeding may underestimate true inbreeding [34,35]. When the distribution of ROH segments in our study is considered, the major feature is a relatively uniform distribution of the frequency of ROH fragments of small, intermediate and large size, with about one-third of the total number of ROH in each category. Indeed, the frequency of long ROH (larger than 16 Mb) was about 32% in our study, whereas the frequency reported for this ROH length in studies with various other breeds ranged between about 12 and 25% [17,34,35]. The same pattern was observed for the mean length of ROH, which was also larger in our study, and confirm the occurrence of long ROH segments in Ramo Grande, and it is known that long ROH segments reflect the occurrence of recent inbreeding in a population, e.g., over the last three generations [16,34]. These results confirm that recent inbreeding has increased severely in the Ramo Grande breed, possibly as a consequence of the fragmentation of the population by various islands, which inevitably leads to reduced gene flow between groups and a higher within-island relationship.

The level of *F*_ROH_ in our study, computed considering a minimum length of 4Mb for ROH, indicates that, in comparison with other studies that have used the same threshold for *F*_ROH_, Ramo Grande cattle have a higher mean level of inbreeding (*F*_ROH_ = 0.09) when compared with Italian local cattle breeds with a census comparable to Ramo Grande, where the mean *F*_ROH_ was about 0.05 [17]. On the other hand, the mean *F*_ROH_ in Ramo Grande was similar to that currently observed in American Holstein [35]. When a threshold of 8 Mb is used to define *F*_ROH_, the mean for Ramo Grande was 0.079, which is close to the mean reported using the same threshold in Brown Swiss (*F*_ROH_ = 0.074), but higher than the means reported for Fleckvieh, Norwegian Red and Tyrol Grey (mean *F*_ROH_ ranging from 0.019 to 0.036) [22].

The distribution of *F*_ROH_ for the studied population indicates that some individuals have extremely high levels of inbreeding (e.g., above 0.25), which may lead to inbreeding depression and loss of genetic variability in the near future [36,37]. This pattern could be identified with the tools of genomic analyses and would probably go unnoticed with pedigree analyses, given the shallowness of pedigrees in Ramo Grande.

The extent of LD was used to estimate current and past effective population size (*N*_e_), and pairwise values of *r*^2^ were averaged and plotted against genomic distance. We observed that the extent of LD in Ramo Grande is higher in comparison with other European local breeds with similar census [17] but similar to the LD decay of Italian Holstein [17] and German Holstein [38]. Therefore, it can be expected that Ne in Ramo Grande is of similar magnitude to that of Holstein in Europe but higher than for other European local breeds. In our case, a steep increase in Ne of Ramo Grande was observed as we go back in time, which may be due to the fact that the Ramo Grande breed was developed in the archipelago of Azores by colonization using Portuguese local mainland breeds since the 16th century, with occasional inflow of germplasm of cosmopolitan breeds in more recent years, which would inflate Ne. Indeed, the introgression of exotic germplasm, especially of Holstein genes, into Ramo Grande, has been shown by analyses with microsatellite markers [5], and crossbreeding will inevitably change the pattern of LD and the estimated Ne of a population [39].

We have further investigated the possibility to identify SNP markers that were significantly associated with production traits for which this breed is currently under selection. Even though the number of animals genotyped was not high, the strategy followed in our study of using deregressed EBV allowed us to take advantage of a large number of phenotypic records collected in up to about 4000 animals, thus enhancing the reliability of the detected associations.

We have identified candidate SNP markers for longevity, for age at first calving and for direct genetic effects on calf weight at four months. Longevity corresponds to the length of productive life and it is, therefore, one of the most important traits in selection of local populations adapted to marginal conditions, where hardiness is crucial. However, longevity is a trait of low heritability, with an estimate in Ramo Grande of 0.07 and estimates in the literature ranging from 0.05–0.14 [40,41]. Therefore, the identification of bona fide markers for genomic selection is of upmost importance, and we have identified new markers associated with this trait. The most significant association was found in BTA1, upstream of *GMNC* gene that is involved in the regulation of DNA replication. The investigation of the interactions of *GMNC* gene with other genes showed a significant enrichment of genes related with cell cycle and cell development, and with regulation of gene expression. SNP variants located in *GMNC* or upstream of this gene have been associated with neuronal diseases in humans, that affect mostly elderly subjects [42,43,44]. To our knowledge, no significant associations between this gene and cattle traits have been reported in the literature. Furthermore we identified other markers located in BTA8 associated with longevity, namely upstream of *PRUNE2* gene and in the intron regions of gene *RAD23B.* Gene *PRUNE2* is involved in the regulation of cell differentiation and survival, and is an important tumour suppressor [45]. In humans, *PRUNE2* has also been associated with neuronal diseases in elderly subjects [42,43,44], while in Nelore cattle the *PRUNE2* gene has been associated with feed intake [46,47]. Gene *RAD23B* is involved in global genome nucleotide excision repair and in humans it has been associated with breast cancer [48] but significant associations in cattle are unknown until now. While considering the gene network generated using these three genes, we observed enrichment of genes involved in DNA repair and in gene expression pathways. A more efficient process of gene repair and control of DNA replication and of gene expression is relevant for a trait such as longevity and therefore more research should be devoted to the role of the identified variants and their corresponding candidate genes in a trait of major importance in all cattle breeds, such as longevity.

We have further identified, although with a lower degree of significance, SNPs significantly associated with age at first calving and with direct genetic effects for calf weight at four months. In relation with age at first calving, our estimate of heritability (0.20) is within the wide range of heritability estimates that have been reported, between 0.02 and 0.47 [49,50,51], and we found a significant association with a SNP located upstream of gene *AQP5*, in a potential regulatory region. The protein encoded by this gene forms a water specific channel [52], playing an important role in water homeostasis which is important for proper functioning of all body fluids, including reproductive structures. Aquaporin 5 protein, along with other aquaporins, namely aquaporin 1 and aquaporin 9 (also involved in *AQP5* gene interaction network) are crucial proteins required to ensure the proper follicular development of the ovary [53], which is crucial for reproductive development and the ability to produce offspring at an early stage. In cattle QTLdb [54] we found a significant association between age at first calving and SNPs located in *STAT5A* [55], and this gene is involved in the same pathway as *ZNF395* [56], which in co-expressed with *AQP5* [57]. This is the first study that identifies *AQP5* as being significantly associated with a trait related with reproductive development and that is functionally associated with a previously identified QTL. Finally, we have studied the trait direct genetic effects for calf weight at four months, for which our estimate of heritability was 0.22, in close agreement with the mean heritability estimate of 0.24 for direct genetic effects for weaning weight [58]. We have further identified significant associations between this trait and SNPs located in introns of two genes, i.e., *KCNMA1* and *CTNNA2*. While *KCNMA1* gene encodes for a protein that forms the potassium channel [59], *CTNNA2* encodes for a protein that may function as a linker between cadherin adhesion receptors and the cytoskeleton to regulate cell-cell adhesion and differentiation in the nervous system [60]. In humans, variants located in *KCNMA1* have consistently been associated with obesity [61,62,63], and *CTNNA2* has been associated with plasma levels of vitamin K [64], that plays a crucial role in heart diseases and with cardiovascular measures of the autonomic tone [65]. The functional analysis of the significant associations found for age at first calving and for direct genetic effects on calf weight at four months, along with the associations reported in the literature, require further analyses for these variants in the future.

## 5. Conclusions

This study reports for the first time the use of genomic information for the maintenance and selection of Ramo Grande cattle, a breed with small census and fragmented dispersion in the archipelago of Azores. The obtained results highlight that the levels of inbreeding currently observed are high in comparison with other European local breeds, and some sires are carriers of very large ROH segments, which may compromise their performance and use. These results are crucial to improve the on-going program aimed at maintaining genetic diversity of this population, in collaboration with local breeders. The analyses of linkage disequilibrium indicate that the estimated effective population size several generations ago is very high, which probably reflects the inflow of exotic germplasm in Ramo Grande in the past. Our results support the existence of various genetic markers associated with longevity, age at first calving and direct genetic effects on calf weight at four months, which are functionally relevant and important for a hardy breed such as Ramo Grande. To our knowledge, these associations have not been previously identified in cattle, and they should be further investigated to confirm that relevant associations with economically important traits also exist in other cattle breeds. Overall, our study confirms the usefulness of genomic information to enhance the genetic management of local populations, both in terms of conservation of their genetic diversity and selection for production efficiency, including adaptation traits.

## Figures and Tables

**Table 1:** Descriptive statistics of the number (N) and the frequency distribution of ROH in different length categories (Mb) in Ramo Grande.

ROH Length Categories	Number of ROH	Frequency (%)
4–8	220	34.4
>8–16	217	33.9
>16	202	31.6

**Table 2:** Descriptive statistics for runs of homozygosity (ROH).

Parameter ^1^	Estimate
MN_ROH_	15.2 ± 10.6 (1–45)
*F*_ROH_	0.091 ± 0.073 (0.0009–0.30)
L_ROH_	14.96 ± 11.99 (4–78.3)
SNPs	124 ± 98.84 (40–669)

^1^ MN_ROH_: mean number of ROH per individual; *F*_ROH_: mean ROH-based individual inbreeding coefficient for ROH > 4 Mb; L_ROH_: average length of ROH in Mb; SNPs: number of single nucleotide polymorphisms (SNPs) involved in ROH. For all estimates the standard deviation is shown as well as the minimum and maximum values (shown in brackets).

**Table 3:** Descriptive statistics (*n* = number of records; $\bar{x}$ = mean; $s_{p}^{2}$ = phenotypic variance) and estimated genetic parameters (*h*^2^ = heritability of direct effects; *m*^2^ = heritability of maternal effects; *r_AM_* = correlation between direct and maternal genetic effects; *c*^2^ = proportion of variance due to permanent environmental effects) for the traits analyzed.

Trait	*n*	$\bar{x}$	$s_{p}^{2}$	*h* ^2^	*m* ^2^	*r_AM_*	*c* ^2^
Type score	3782	78.67	21.913	0.281			
Age at first calving	2169	841	29349	0.202			
Calving interval	9845	418	11,554.7	0.073			0.069
Longevity	1493	102.94	1020.2	0.079			
No. calvings	1493	5.22	5.5435	0.146			
Carcass weight/day (g)	2728	477.5	16,401.6	0.588	0.132	−0.512	0.001
Live weight at 4 mo. (kg)	661	159.4	1007	0.272	0.171	−0.230	0.001
Temperament	1062	2.476	0.4645	0.378			

**Table 4:** List of SNPs significantly associated with longevity (LONG), age at first calving (AFC) and direct genetic effects for calf weight at 4 months (DCW4) identified by GWAS, with corresponding slope (Beta) and standard error (SE), proportion of the variance explained by the SNP (PVE), chromosome location CHR—Position), significance (*p*-value), type of location (Type) and associated genes (Feature; NA: not applicable).

Trait	SNP Name	Beta	SE	PVE	CHR	Position	*p*	Type	Feature
LONG	*rs110680473*	2.64	0.53	0.37	1	79190806	9.53 × 10^−7^	intergenic	NA
	*rs110454414*	2.08	0.58	0.23	1	82440177	2.14 × 10^−6^	intergenic	NA
	*rs110789800*	2.25	0.49	0.33	1	76375060	7.28 × 10^−6^	upstream gene variant	*GMNC*
	*rs110554326*	2.62	0.59	0.32	1	85965329	9.47 × 10^−6^	Intergenic	NA
	*rs41635194*	1.84	0.43	0.30	1	86011556	2.54 × 10^−5^	Intergenic	NA
	*rs41597797*	2.52	0.59	0.30	8	97033809	3.20 × 10^−5^	intron variant	*RAD23B*
	*rs42443533*	−0.74	0.18	0.29	1	107758823	3.42 × 10^−5^	Intergenic	NA
	*rs42452113*	−0.56	0.14	0.29	1	104139607	4.62 × 10^−5^	Intergenic	NA
	*rs110589031*	1.49	0.36	0.29	8	52721756	4.84 × 10^−5^	upstream gene variant	*PRUNE2*
	*rs43096527*	1.49	0.36	0.29	8	52655614	4.84 × 10^−5^	Intergenic	NA
	*rs41609616*	1.91	0.47	0.28	8	96631094	4.96 × 10^−5^	Intergenic	NA
AFC	*rs29021075*	−34.06	8.15	0.29	5	29913746	2.72 × 10^−5^	upstream gene variant	*AQP5*
DCW4	*rs110789957*				28	33061971	1.51 × 10^−5^	Intron variant	*KCNMA1*
	*rs110783886*	−4.00	0.92	0.31	11	62811507	2.18 × 10^−5^	Intergenic	NA
	*rs41663416*	−1.64	0.38	0.30	11	55575538	4.21 × 10^−5^	Intron variant	*CTNNA2*
	*rs41627532*	−1.53	0.37	0.29	13	29432282	5.25 × 10^−5^	Intergenic	NA
	*rs110789957957*	3.26	0.80	0.29	28	33061971	1.51 × 10^−5^	Intron variant	*KCMA1*

**Table 5:** Pathway enrichment analysis for genes of the Longevity network. The table shows the 10 most relevant pathways sorted by *p*-value.

Pathway Name	Entities				Reactions	
	Found ^a^	Ratio ^b^	*p* ^c^	FDR ^d,^*	Found ^e^	Ratio ^f^
Formation of Incision Complex in GG-NER	13/199	0.01	1.11 × 10^−^^16^	1.55 × 10^−^^14^	10/10	8.19 × 10^−^^4^
Global Genome Nucleotide Excision Repair (GG-NER)	13/251	0.012	1.11 × 10^−^^16^	1.55 × 10^−^^14^	18/20	2 × 10^−^^3^
Dual Incision in GG-NER	10/61	0.003	5.55 × 10^−^^16^	5.16 × 10^−^^14^	3/3	2.46 × 10^−^^4^
Nucleotide Excision Repair	13/331	0.016	1.67 × 10^−^^15^	1.17 × 10^−^^13^	34/36	3 × 10^−^^3^
Dual incision in TC-NER	9/112	0.006	1.29 × 10^−^^13^	7.21 × 10^−^^12^	6/6	4.92 × 10^−^^4^
Transcription-Coupled Nucleotide Excision Repair (TC-NER)	9/187	0.009	1.20 × 10^−^^11^	5.53 × 10^−^^10^	16/16	1 × 10^−^^3^
DNA Repair	13/1202	0.06	1.46 × 10^−^^8^	5.17 × 10^−^^7^	40/326	2.7 × 10^−^^3^
DNA Damage Recognition in GG-NER	5/48	0.002	1.48 × 10^−^^8^	5.17 × 10^−^^7^	5/5	4.10 × 10^−^^4^
Formation of TC-NER Pre-Incision Complex	7/130	0.006	5.72 × 10^−^^8^	1.77 × 10^−^^6^	7/7	5.74 × 10^−^^4^
Gap-filling DNA repair synthesis and ligation in TC-NER	5 / 66	0.003	7.12 × 10^−^^8^	1.99 × 10^−^^6^	2/2	1.64 ×10^−^^4^

^a^ Entities found: number of curated molecules that are common between the submitted list of genes and the pathway identified. ^b^ Entities ratio: proportion of Reactome pathway molecules for the respective pathway that are in the list of molecules of the input list of genes. ^c^ Entities *p*-value: The result of the statistical test for over-representation, for molecules of the respective pathway. ^d^ Entities FDR: false discovery rate, corrected over-representation probability. ^e^ Reactions found: the number of reactions in the pathway that contain molecules of the type of the selected pathway. ^f^ Reactions ration: proportion of Reactome reactions represented by the respective pathway.

**Table 6:** Pathway enrichment analysis for genes of the age at first calving network. The following table shows the 10 most relevant pathways sorted by *p*-value.

Pathway Name	Entities				Reactions	
	Found ^a^	Ratio ^b^	*p* ^c^	FDR ^d,^*	Found ^e^	Ratio ^f^
Passive transport by aquaporins	12/21	1 × 10^−3^	1.1 × 10^−16^	1.2 × 10^−14^	6/8	6.6 × 10^−4^
aquaporin-mediated transport	12/137	7 × 10^−^^3^	1.1 × 10^−16^	1.2 × 10^−14^	15/25	2 × 10^−3^
Transport of small molecules	14/1841	9.2 × 10^−3^	5.7 × 10^−8^	5.4 × 10^−7^	24/437	3.6 × 10^−3^
Transport of glycerol from adipocytes to the liver by aquaporins	2/3	1.49 × 10^−4^	6.6 × 10^−6^	3.7 ×10^−4^	2/2	1.6 × 10^−4^
Vasopressin regulates renal water homeostasis via aquaporins	4/121	6 × 10^−3^	1.5 × 10^−5^	6.6 × 10^−4^	7/15	1 × 10^−3^
Ca2+ activated K+ channels	1/17	8.5 × 10^−4^	2.1 × 10^−3^	3.5 × 10^−3^	1/3	2.5 × 10^−4^
Reversible hydration of carbon dioxide	1/17	8.5 × 10^−4^	2.1 × 10^−3^	3.5 × 10^−3^	2/8	6.5 × 10^−4^
Erythrocytes take up oxygen and release carbon dioxide	1/21	1 × 10^−3^	2.6 × 10^−3^	3.5× 10^−3^	1/6	4.9 × 10^−4^
GLI proteins bind promoters of Hh responsive genes to promote transcription	1/23	1 × 10^−3^	2.8 × 10^−3^	3.5 × 10^−3^	2/4	3.3 × 10^−4^
Nuclear signaling by ERBB4	2/224	1.1 × 10^−3^	3.1 × 10^−3^	3.5 × 10^−3^	2/34	3 × 10^−3^

^a^ Entities found: number of curated molecules that are common between the submitted list of genes and the pathway identified. ^b^ Entities ratio: proportion of Reactome pathway molecules for the respective pathway that are in the list of molecules of the input list of genes. ^c^ Entities *p*-value: The result of the statistical test for over-representation, for molecules of the respective pathway. ^d^ Entities FDR: False discovery rate, corrected over-representation probability. ^e^ Reactions found: the number of reactions in the pathway that contain molecules of the type of the selected pathway. ^f^ Reactions ration: proportion of Reactome reactions represented by the respective pathway.

**Table 7:** Pathway enrichment analysis for genes of the direct genetic effects for calf weight at four months network. The following table shows the 10 most relevant pathways sorted by *p*-value.

Pathway Name	Entities				Reactions	
	Found ^a^	Ratio ^b^	*p* ^c^	FDR ^d,^*	Found ^e^	Ratio ^f^
Ca2+ activated K+ channels	4/19	9.2 × 10^−4^	1.23 × 10^−7^	5.22 × 10^−5^	1/3	2.4 × 10^−4^
cGMP effects	4/21	1 × 10^−^^3^	1.82 × 10^−7^	5.22 × 10^−5^	1/4	3.2 × 10^−4^
Nitric oxide stimulates guanylate cyclase	4/70	3 × 10^−^^3^	2.08 × 10^−5^	4 × 10^−^^3^	1/7	5.5 ×10^−4^
Potassium channels	4/128	6 × 10^−^^3^	2.11 × 10^−4^	0.03 × 10^−^^3^	1/19	1 × 10^−^^3^
Thromboxane signalling through TP receptor	4/61	3 × 10^−^^3^	3.76 × 10^−4^	4.3 × 10^−^^3^	8/8	6.3 × 10^−4^
Signal amplification	4/102	5 × 10^−^^3^	2 × 10^−^^3^	1.6 × 10^−^^3^	10/19	1 × 10^−^^3^
Platelet homeostasis	5/252	1.2 × 10^−^^3^	3 × 10^−^^3^	2 × 10^−^^3^	2/30	2 × 10^−^^3^
Drug resistance in ERBB2 TMD/JMD mutants	1/5	2.4 × 10^−4^	1.1 × 10^−^^3^	3.7 × 10^−^^3^	1/1	7.9 × 10^−5^
Resistance of ERBB2 KD mutants to trastuzumab	1/5	2.4 ×10^−4^	1.1 × 10^−^^3^	3.7 × 10^−^^3^	1/1	7.9 × 10^−5^
Resistance of ERBB2 KD mutants to neratinib	1/5	2.42 × 10^−4^	1.1 × 10^−^^3^	3.7 × 10^−^^3^	1/1	7.9 × 10^−5^

^a^ Entities found: number of curated molecules that are common between the submitted list of genes and the pathway identified. ^b^ Entities ratio: proportion of Reactome pathway molecules for the respective pathway that are in the list of molecules of the input list of genes. ^c^ Entities *p*-value: The result of the statistical test for over-representation, for molecules of the respective pathway. ^d^ Entities FDR: false discovery rate, corrected over-representation probability. ^e^ Reactions found: the number of reactions in the pathway that contain molecules of the type of the selected pathway. ^f^ Reactions ration: proportion of Reactome reactions represented by the respective pathway.

## Supplementary Materials

The following are available online at https://www.mdpi.com/2076-2615/10/6/1089/s1. Table S1: Number of ROH segments per chromosome, Table S2: Summary statistics of ROH length (Mbps) per chromosome.
