# Supplementary material for: Genomic Tools for the Conservation and Genetic Improvement of a Highly Fragmented Breed—The Ramo Grande Cattle from the Azores
Source: Animals (Basel). 2020 Jun 24;10(6):1089. doi: 10.3390/ani10061089 (PMC7341246; doi:10.3390/ani10061089)
Supplement: Supplementary file 1 [file animals-10-01089-s001.pdf]

S1 Table- Number of ROH segments per chromosome.

Chromosome	Total Count of ROH Segments
1	30
2	22
3	26
4	33
5	33
6	39
7	26
8	30
9	21
10	25
11	30
12	23
13	19
14	25
15	24
16	19
17	19
18	22
19	20
20	12
21	18
22	22
23	12
24	13
25	13
26	28
27	10
28	13
29	12
Grand Total	639

S2 Table – Summary statistics of ROH length (Mbps) per chromosome.

Chromosome	Mean	Min	Max	Stdev
1	15,58769547	4,139583	52,837796	12,53992319
2	11,49754395	4,184694	28,552765	6,850616767
3	16,10925592	4,144932	78,304324	17,36421542
4	17,07648576	4,043861	67,871682	14,80494059
5	18,4749803	5,283996	54,843229	12,08825233
6	18,15638618	4,377729	51,374175	13,23605148
7	15,87126081	4,341681	51,698911	13,02790834
8	22,40710187	4,291997	53,172577	13,99734296
9	12,9276191	4,822196	37,491164	9,052265869
10	12,93534056	4,142529	40,169058	9,097745465
11	17,3013354	4,210089	58,507927	13,51843766
12	13,2998557	4,032933	70,143409	16,12082354
13	15,88417816	4,09097	60,619246	16,64171289
14	14,64778696	4,280308	48,397142	10,74701343
15	12,44985363	5,070148	51,668992	10,53061347
16	13,30724321	4,213137	36,679059	8,491541351
17	16,98839295	5,911399	49,134195	12,07306859
18	14,34071791	5,378589	57,368842	11,69819217
19	12,5913727	4,187886	25,424659	6,633825869
20	11,54404775	4,181933	50,946867	12,71363581
21	10,4362505	4,087774	39,968973	8,301042487
22	14,05612723	6,03456	46,589201	9,5354507
23	10,27638058	4,006504	34,214953	8,790677744
24	14,94435985	4,27848	51,937833	12,68065137
25	8,834092077	4,047632	17,689957	4,497647401
26	15,92426921	4,650936	37,439951	8,417221642
27	10,6097734	4,300144	27,540875	7,88553509
28	9,504696692	4,256419	24,943762	6,476384667
29	15,42706475	5,132827	35,292474	10,96665025
